# Enrichment of circulating head and neck tumour cells using spiral microfluidic technology

**DOI:** 10.1038/srep42517

**Published:** 2017-02-15

**Authors:** Arutha Kulasinghe, Thao Huynh Phuoc Tran, Tony Blick, Ken O’Byrne, Erik W. Thompson, Majid E. Warkiani, Colleen Nelson, Liz Kenny, Chamindie Punyadeera

**Affiliations:** 1 The School of Biomedical Sciences and Institute of Health and Biomedical Innovation, Queensland University of Technology, Kelvin Grove, QLD, Australia; 2 Translational Research Institute, Woolloongabba, QLD, Australia; 3 Translational Cell Imaging Queensland, Institute of Health and Biomedical Innovation, Queensland University of Technology, Qld, Australia; 4 University of Melbourne, Department of Surgery, St Vincent’s Hospital, Melbourne, Australia; 5 School of Mechanical and Manufacturing Engineering, Australian Centre for NanoMedicine, University of New South Wales, Sydney, Australia; 6 Australian Prostate Cancer Research Centre - Queensland, Institute of Health and Biomedical Innovation, Queensland University of Technology, Princess Alexandra Hospital, Translational Research Institute Brisbane, Australia; 7 School of Medicine, University of Queensland; Royal Brisbane and Women’s Hospital, Brisbane; Central Integrated Regional Cancer Service, Queensland Health, Queensland, Australia

## Abstract

Whilst locoregional control of head and neck cancers (HNCs) has improved over the last four decades, long-term survival has remained largely unchanged. A possible reason for this is that the rate of distant metastasis has not changed. Such disseminated disease is reflected in measurable levels of cancer cells in the blood of HNC patients, referred to as circulating tumour cells (CTCs). Numerous marker-independent techniques have been developed for CTC isolation and detection. Recently, microfluidics-based platforms have come to the fore to avoid molecular bias. In this pilot, proof of concept study, we evaluated the use of the spiral microfluidic chip for CTC enrichment and subsequent detection in HNC patients. CTCs were detected in 13/24 (54%) HNC patients, representing both early to late stages of disease. Importantly, in 7/13 CTC-positive patients, CTC clusters were observed. This is the first study to use spiral microfluidics technology for CTC enrichment in HNC.

Head and neck cancers (HNCs) account for the 7^th^ most common tumour type globally. Whilst there have been improvements in locoregional control of HNC, distant metastasis remains a challenge^1^. Circulating tumour cells (CTCs), are rare cancer cells shed into circulation, representing metastatic seeds and providing a window into metastasis. CTCs have the potential to provide critical information on the metastatic cascade, tumour heterogeneity and chemoresistance^2^,^3^,^4^,^5^. Whilst CTCs have been well studied in metastatic breast, prostate and colorectal cancer patients^3^,^6^, the HNC CTC field remains in its infancy^1^,^7^,^8^,^9^. Epidermal growth factor receptor (EGFR), which has been shown to be amplified in HPV-negative tumours compared to HPV-positive tumours, has previously been characterized in HNC CTCs^7^,^9^,^10^,^11^,^12^,^13^,^14^.

CTCs, when present, are extremely rare in comparison to the plethora of white blood cells in circulation^15^. The CellSearch system (Janssen Diagnostics) has FDA-approval for CTC enumeration in a number of tumour types and has been used to demonstrate the clinical relevance of EpCAM-positive CTCs^6^. This system has been robust in CTC enumeration through marker based CTC capture^6^,^16^. Numerous marker-based assays using microfluidic technologies are available and have been previously reported^17^,^18^. However, it has been shown that affinity based platforms have limitations due to poor sensitivity^19^,^20^. This is further compounded by varying levels of cell surface marker expression, CTC heterogeneity and specific processes such as epithelial-to-mesenchymal transition (EMT)^1^,^21^,^22^. The current view is that CTCs can show an intermediate EMT phenotype^2^,^20^,^23^ as well as express varying degrees of other markers such as PD-L1, CXCR4 and Plastin-3^2^,^24^. Based on the heterogeneity found among CTCs, compounded with sometimes low-EpCAM expression, critical subpopulations may not be detected. Therefore, various marker-independent CTC enrichment strategies need to be tailored for each tumour type^18^,^19^,^20^. Marker-independent enrichment relies on the physical properties of CTCs such as size, deformability, charge and density^1^,^15^,^19^,^25^,^26^.

Recently, there has been a demand in the field for the isolation of viable CTCs on which to perform functional assays. There has been a shift toward methodologies that allow for (i) marker-independent CTC capture (ii) CTC propagation (iii) minimal pre-enrichment processing and (iv) processing larger volumes of blood in a short time period^1^,^2^,^27^,^28^,^29^. To encompass the above, the spiral microfluidics technology developed by Warkiani *et al*.^15^,^27^,^30^,^31^,^32^. was evaluated. The spiral chip is a marker-independent and high-throughput device that utilises hydrodynamic forces present in curvilinear microchannels for size-based cell sorting^15^,^27^. The spiral chip has previously been reported for use in breast and lung cancers^32^. CTC separation from lysed whole blood occurs based on CTC deformability and size. In this study, we enriched blood from 24 HNC patients using the spiral microfluidics platform and enumerated CTCs using immunocytochemistry. Our evaluation is the first study using the spiral technology for CTC enrichment from patients with HNCs, and shows positive results. Future, multi-centre trials are required and warranted before this technology can be implemented in a clinical setting.

## Results

### Spiral Chip enrichment

**Figure 1:**
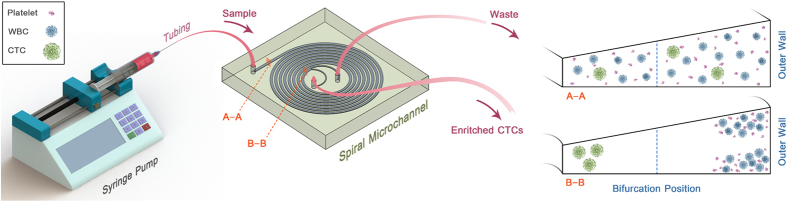
Spiral microfluidic chip setup.

The spiral chip utilizes the inherent Dean vortex flows present in curvilinear microchannels under continuous flow, along with inertial lift forces, which focus the CTCs near the microchannel inner wall while driving the smaller hematologic cells toward the microchannel outer wall, allowing an efficient separation at the outlet (Fig. 1). The cells are separated as a function of deformability and size, as cells occupy different lateral positions away from the walls of the microchannel, allowing for separation at the bifurcation position^27^. Typically, cells larger than 14 μm flow into the “CTC outlet” and cells smaller than this size flow into the “waste outlet.”

### Recovery Studies

**Figure 2:**
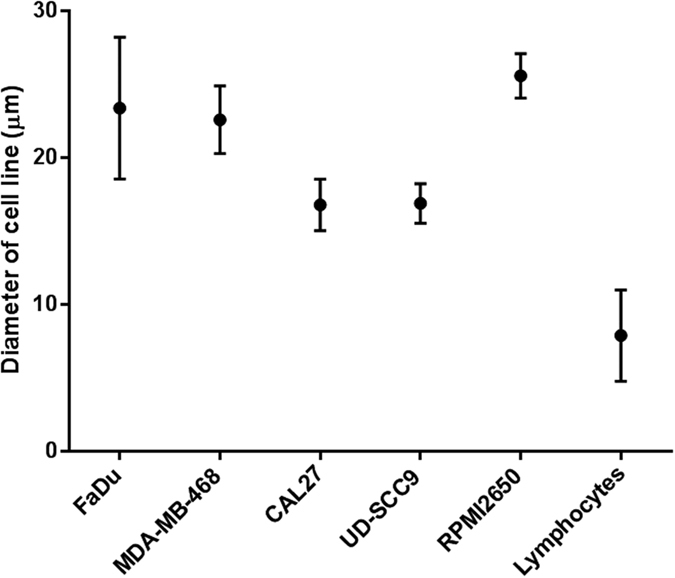
The mean diameter and standard deviation of 5 human cell lines and normal human lymphocytes.

**Figure 3:**
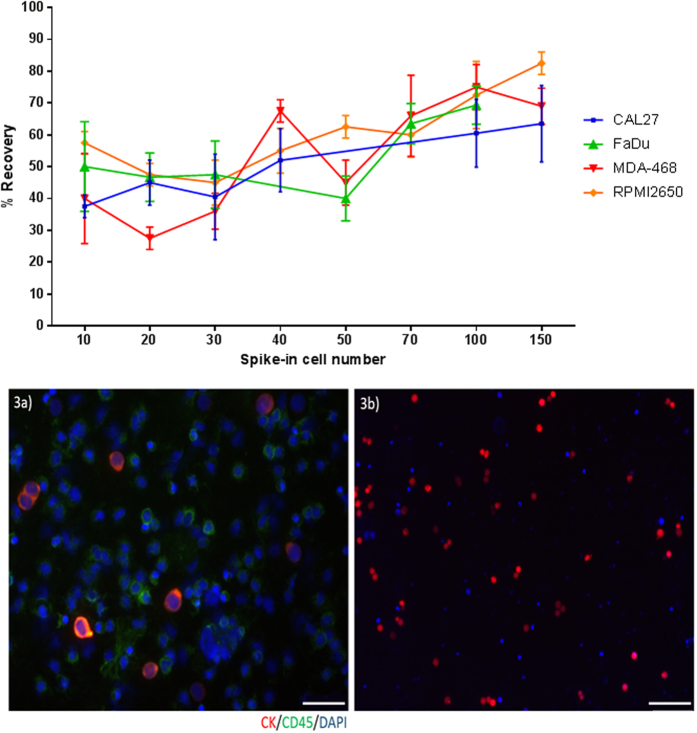
(**i**) The mean % recovery and standard deviation for spiked cell lines (n = 3). (**ii**, a) Presentation of spike-in and recovered tumour cells FaDu cells into blood of a normal healthy volunteer. Tumour cells: cytokeratin (red), DAPI (blue) White blood cells: CD45 (green), DAPI (blue). Scale bar represents 10 μm (b) RFP-labelled tumour cells from whole blood. Scale bar represents 50 μm.

**Figure 4:**
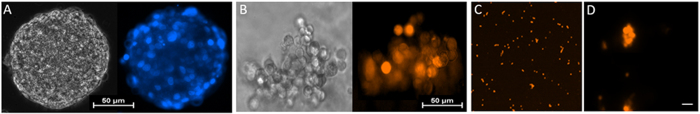
Day 24, Bioreactor grown (**A**) FaDu spheroid (**B**) MDA-MD-468 (cell cluster). (**C**) MDA-MD-468 Spiral sorted single and cell clusters (**D**) 600x image of Spiral sorted cell cluster. Scale bar represents 10 μm.

FaDu, CAL27, RPMI2650 and MDA-MB-468 cell line cells, which all have a mean diameter above 15 μm (Fig. 2), were used for spike-in experiments. The percentage recovery of spiked-in cells (Fig. 3i) ranged from 60–70% when experiments were performed with 100–150 cells spiked into 10 mL blood, trending to lower recover rates (40–60%) when less cells were spiked in. The overall recovery of the spiked cell lines were comparable to each other. We also assessed the decrease in background leukocytes and recovery of cancer cells after repeat passages through the spiral chip. When the samples were collected from the “CTC outlet” and run through the chip again, a 30% decrease in leukocytes was observed, however there was a 10–15% decrease in cancer cell recovery which was accounted for in the dead volumes of the setup. More than 1 repeat run did not further purify the sample. Single cells and cell clusters were able to be enriched by the spiral chip (Fig. 3ii). Similarly, bioreactor-cultured spheroids could also be enriched by the spiral chip (Fig. 4).

### Patient Characteristics

A total of 24 patients and 8 normal healthy controls were included in this study. Patient characteristics are shown in Table 1. The median age was 62 years (range 36–89); including men (n = 22), women (n = 2). The most distinctive histology was mucosal oral cavity SCC (63%). The majority of patients presented with tumour grading T2/T3 (71%) and either no nodal spread N0 (50%) or advanced nodal spread N2a-c (50%). Patients were assessed for distant metastases by CT/PET-CT and MRI scans. All patients were found to be radiographically M0 upon assessment. CTC status was determined at the same time point. The clinicopathological patient findings are presented in Table 2.

### Enrichment of CTCs from controls and patients

**Figure 5:**
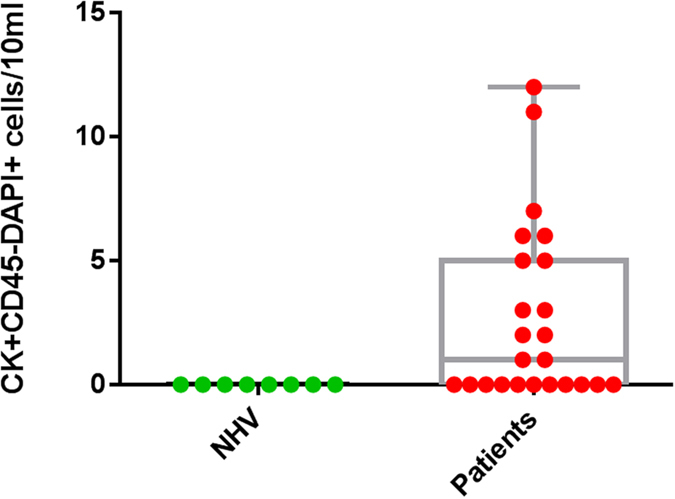
Box plot summary indicating the range of CTCs (pan-CK + CD45-DAPI + ) cells/10 ml. The box plot presents the median and all individual data points. NHV; normal healthy volunteers.

**Figure 6:**
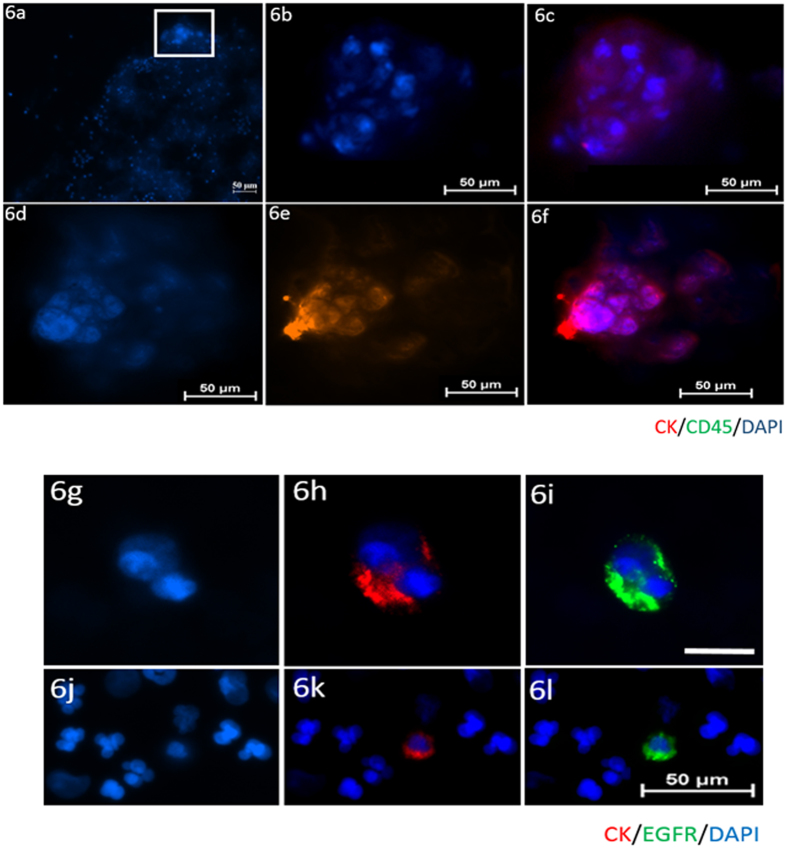
Patient “micro_01, micro_06”; CTC clusters detected in the blood. (**a**) 100x magnification showing the CTC cluster in the top of the cytospot. 1000x images of (**b**) DAPI (**c**) Cytokeratin/DAPI composite (**d**) DAPI (**e**) Cytokeratin (**f**) Cytokeratin/DAPI composite. Scale bar represents 50 μm. Figure 6 continued. Patient “micro_12”; CTC doublet detected in blood. (**g**) DAPI (**h**) Cytokeratin/DAPI composite (**i)** EGFR/DAPI composite. Patient “micro_03” single CTCs detected in blood. (**j**) DAPI (**k**) Cytokeratin/DAPI composite (**l**) EGFR/DAPI composite. Scale bar 10 μm (**g–i**). Scale bar 50 μm (**j–l**).

**Figure 7:**
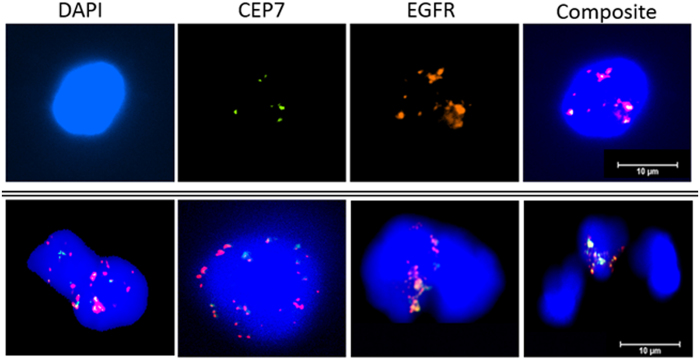
EGFR gene amplification in CTCs derived from head and neck cancer patients. Dual colour FISH assay probes for EGFR (red) and chromosome seven (CEP7, green). Row 1 shows individual stains for DAPI, CEP7, EGFR and the composite image (DAPI, CEP7, EGFR). Row 2 shows further patient CTC composite images showing EGFR amplification. Scale bar represents 10 μm.

10 mL blood samples were obtained from 8 healthy volunteers and 24 head and neck cancer patients and processed using the spiral chip. All samples were enumerated for CTCs. Putative CTCs were identified on the stained cytospots as positive for: pan-CK, DAPI and negative for CD45. Further characterisation of head and neck CTCs was performed by EGFR ICC and DNA FISH. Leukocytes were identified as positive for CD45, DAPI and negative for pan-CK. No CTC-like events were observed in the 8 normal healthy volunteers (Fig. 5). CTCs were detected in 13/24 (54%) (Range 1–12 CTCs/10 ml blood sample) of the HNC patients screened in the study. CTC clusters were readily detectable in 7/13 (53.8%) of the CTC-positive patients (Fig. 6). The CTCs were further molecularly characterised and found to have EGFR gene amplifications (Fig. 7). Further observations made in the patient samples include double positive pan-CK and CD45 cells in 2/20 samples and 27 CK-negative, CD45-negative cells in 1 patient presenting with a CTC cluster. These events were not considered for enumeration in the study.

## Discussion

HNC patients often present with advanced metastatic spread, predominantly to the lungs, liver and bone^1^,^33^. The ability to assess “intact” metastatic precursors in the form of tumour cells in blood holds remarkable potential as a liquid biopsy. The number of CTCs, and molecular and genetic changes in the CTCs could be monitored in patients following therapy and non-responders determined at an earlier time point^4^,^34^,^35^. Moreover, patients whose disease will potentially respond to specific therapies could be identified. Recent studies have shown that PD-L1 is frequently expressed on CTCs and that these are present in patients where anti-PDL1 therapies could be effective^24^,^36^,^37^. Numerous CTC enrichment platforms have been developed in recent years, each with its own advantages and limitations^1^,^21^,^38^,^39^,^40^. In this study, we tested and evaluated the spiral microfluidics chip for CTC enumeration and characterization. This platform has features which are of value to clinical research; CTCs are found in suspension and not immobilized allowing for functional assays and CTC clusters can be readily isolated with a leukocyte depletion of up to 4 log^15^. Limitations of the spiral technology are that “small CTCs” or CTCs ( < 12 μm) in size will be lost^15^,^41^.

Four cell lines representing a mean cell diameter range between 16–28 μm were chosen for the spiking experiments to get a decent coverage of known head and neck cancer cells. In our hands, the percentage recovery of the spiral chip is similar to that of other microfluidic CTC isolation platforms^19^,^21^. By repeat passages of the spiral chip, a decrease of 30% background leukocytes was found at the expense of 10–15% recovery. However, repeated passages were not performed with patient samples so as not to risk losing CTCs. The range of detected CTCs in HNC patients remains low^1^,^13^,^42^,^43^. Moreover, it was observed that the same population of leukocytes was present after 2 or more repeat runs and this could not be further purified. Furthermore, the spiral chip successfully sorted for cell clusters and tumour spheroids in the CTC outlet.

This pilot study evaluated the use of a spiral chip for CTC enrichment in HNCs. HNC patients ranged from early stage (T1N0M0) to advanced stage patients (T4N2b). Patients with early stage disease present with low rates of distant metastasis and a favourable prognosis. Whilst patients with locally advanced disease are more likely to have CTCs^44^. CTCs were observed in 50% of the HNC samples, ranging from 1–12CTCs/10 mL sample. This finding is consistent with previously published studies using marker-independent enrichment platforms^7^,^13^,^45^. Notably, post spiral enrichment involves transferring rare cells onto a glass slide and immunocytochemistry which can lead to cell loses^34^. Therefore, the actual CTC count per HNC patient could be much higher.

**Figure 8:**
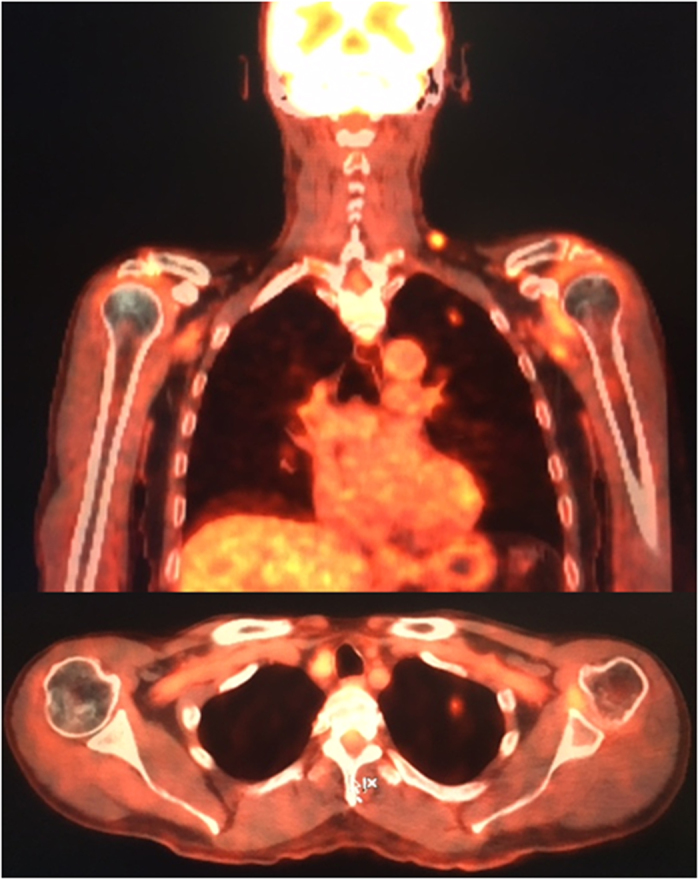
A FDG-PET study from a patient presenting with CTCs and had progressive disease to the lungs showing an 8 × 5 mm nodule in the left upper lobe.

Over half of the CTC positive patients presented with clusters of cancer cells. These multicellular CTC clusters have shown 23–50 fold increased metastatic potential compared to single CTCs in breast cancers and associations with adverse clinical outcomes^1^,^46^,^47^,^48^,^49^, however the clinical significance in HNC is yet to be determined^1^,^50^,^51^,^52^. Moreover, these cell clusters have been reported to have a shorter circulation half-life in blood, consistent with rapid entrapment within the capillaries of distant organs^46^. Initial studies have shown that CTC clusters are potent initiators of distant organ metastasis, highlighting the need to develop strategies which disrupt cell-cell or cell-matrix adhesions in these clusters to reduce metastatic potential^53^. Further investigation is required to determine the clinical implications of CTC clusters in HNC patients. Importantly, CTCs were found upon presentation to clinic in 3/3 patients presenting with queried lung lesions by the MDT clinic (Fig. 8). These lung lesions were to be investigated by ultrasound guided fine-needle biopsy to determine the extent of distant metastasis. In these patients, a window for treatment escalation could become a possibility if the presence of CTCs predicts for patients at-risk of developing metastasis. Further studies assessing CTCs during the course of treatment and timing of blood sampling are warranted to investigate the role of monitoring CTCs in HNC^50^.

## Conclusion

We present the first study in HNCs using a microfluidics based approach for CTC enrichment. 24 HNC patient blood samples were enriched using the spiral microfluidics chip. We were able to identify single and clusters of CTCs and further characterise these cells by ICC and DNA FISH. This study highlights the use of the spiral microfluidic technology in HNC and provides future clinical use in the field of CTCs.

## Materials and Methods

### Patient cohort

Ethics approval was obtained from the Metro South and North Health Service District Human Research Ethics Committee in accordance with the National Health and Medical Research Council’s guidelines (HREC/12/PHAH381) to collect blood from the Royal Brisbane and Women’s Hospital (RBWH). All methods were performed in accordance with these ethical guidelines and regulations. This study also has Queensland University of Technology ethics approval (1400000617). After written informed consent was obtained from the participants, 10 mL blood samples were collected from patients presenting to the HNC clinic.

### Cell lines and culture

The cell lines FaDu (ATCC^-^HTB43), CAL27 (ATCC^-^CRL2095), RPMI2650 (ATCC^-^CCL30) were obtained from ATCC (Manassas, VA). UD-SCC9 HNC cells (ATCC^-^CRL1629) were a generous gift from A/Prof Nick Saunders (UQDI). The MDA-MB-468 breast cancer cells were originally from the ATCC transferred from the Lombardi Cancer Center and fluorescently tagged with pCherryN1, a modification of the Clonetech vector, kindly provided by Dr Alpha Yap, (UQ). Cells were cultured under standard conditions in humidified incubators at 37 °C, 5% CO_2_ in RPMI-1640-Glutamax (Life Technologies, Inc) supplemented with 10% fetal bovine serum (FBS) and 1% Penicillin/Streptomycin (Pen/Strep).

### 3D cell culture (bioreactor)

Cells were spun down to obtain cell pellets post-trypsinization. The cell pellets were resuspended in pre-warmed 1x Happy Cell media (Biocroi Ltd, Dublin, Ireland) supplemented with 10% FBS and 1% Pen/Strep. Optimal seeding densities for the bioreactors were determined by titrating cells from 10,000–500 cells/ml. 1,500 cells/ml, resuspended in 1x Happy Cell media were layered over 2x Happy Cell media in the bioreactor. This gradient ensured that the cells remained in suspension over the incubation periods. The cells were incubated at 37 °C, 5% CO_2_ and imaged every 3 days. To collect the spheroids, the cell suspension was transferred to a 96 well format and an inactivation buffer was used to collapse the hydrogel matrix for 1 hour at 37 °C.

### Spiking of cell lines into bloods collected from normal healthy controls

Blood was collected from normal healthy volunteers in 10 mL EDTA (BD-Plymouth, UK) tubes. The cell lines used for spiking were labelled with CellTracker™ Green (Life Technologies) as per manufacturer’s instructions. The collected blood was spiked with clinically relevant numbers (10–150) of cells within 2 hours of collection. Cell lines for spiking were harvested by trypsinisation for 5 mins at 37 °C and washed in the culture media. The cell suspension was centrifuged at 300 × g for 5 mins, the supernatant removed and cells resuspended in PBS buffer. Cell counts were performed with the Countess II FL automated Cell counter (ThermoFisher Scientific). The appropriate spike-in volume of cells was added to the blood.

### Preparation of blood sample for the spiral Chip

To reduce the cellular components passing through the spiral chip, an initial red blood cell (RBC) lysis was performed, which has been shown to have minimal effects on the recovered cells^15^. Briefly, RBC lysis buffer (Astral Scientifix) was added to the blood sample, after which cells were centrifuged and the pellet resuspended in 10 mL of sheath buffer (1xPBS, 2 mM EDTA, 0.5% BSA).

### Setup and running of the spiral Chip

Tygon^®^ tubing was inserted into the inlet/outlets of the spiral chip, and the inlet tubing connected to a syringe pump. The spiral chip was positioned and fixed onto a phase contrast microscope (Olympus IX71). The outlet tubing was connected to two sterile 15 mL collection BD Falcon tubes. An initial priming run was performed using the sheath buffer at a flow rate of 2.0 mL/min for 5 minutes. To test patient samples, the sample was loaded carefully into a 10 ml syringe (Terumo) and pumped through the spiral chip using the syringe pump at a flow rate of 1.7 mL/min for all experiments. The outputs were collected and spun down at 300× g for 5 mins.

### Immunostaining

Prelabeling and immunocytochemistry (ICC) were used for the detection of spiked tumour cells and CTCs enriched by the spiral microfluidic chip. Enriched cells were cytospun onto a glass slide and CTCs identified by ICC using the CellSearch antibody cocktail (Cytokeratin/CD45/DAPI; Janssen Diagnostics). Cells were further characterised for cell surface EGFR using an anti-EGFR antibody (AY13, BioLegend, San Diego). Briefly, the cytospun sample was air dried overnight and incubated with a mixture of CellSearch reagents (20 ul staining reagent, 20 μl DAPI, 20 μl permeabilization buffer, 20 ul fixation buffer in 120 μl PBS). The slide was incubated for 1 hour at room temperature, washed 3 times in PBS, coverslipped and imaged under a fluorescent microscope. The CellSearch criteria for CTCs were used for CTC identification: intact, cytokeratin-positive, DAPI-positive, CD45-negative cells that were larger than 4 um were considered a CTC. Cells staining positive for CD45 and DAPI and negative for pan-cytokeratin were determined to be white blood cells.

### DNA Fluorescence *in situ* hybridization

Cytospots were placed in pretreatment solution for 10 mins at 98 °C, and digested with pepsin for 5 mins at 37 °C in the Dako hybridiser. After dehydration through a graded ethanol series (70%, 85%, 96%), dual colour, dual target DNA FISH assays were done with *EGFR*/CEN-7 FISH probe mix (DakoCytomation, Glostrup, Denmark). Cytospots were covered with 10 μL probe solution, sealed with rubber sealant and placed in the Dako Hybridizer for denaturation (5 mins, 82 °C) and hybridisation (18 hours, 45 ^o^C). Post hybridisation stringency washes were performed at 65 ^o^C for 10 mins. After further washing and dehydration steps, the cytospots were air dried and mounting media containing DAPI added. The cytospot was coverslipped and imaged on the Nikon Eclipse Ti inverted microscope fitted with a Nikon digital camera. *EGFR* was visualized as a red signal (tetramethylrhodamine isothiocyanate filter), CEP7 (fluorescein isothiocyanate filter) as a green signal and the nuclei as a blue signal with a DAPI filter. *EGFR* status was scored as the number of *EGFR* signals per nucleus and as the ratio of *EGFR* signals to CEP7 signals.

## Additional Information

**How to cite this article**: Kulasinghe, A. *et al*. Enrichment of circulating head and neck tumour cells using spiral microfluidic technology. *Sci. Rep.* **7**, 42517; doi: 10.1038/srep42517 (2017).

**Publisher's note:** Springer Nature remains neutral with regard to jurisdictional claims in published maps and institutional affiliations.

## Figures and Tables

**Table 1:** Patient demographics (n = 24).

Variables	N
Total	24 (100%)
Gender	
Male	22 (91.7%)
Female	2 (8.3%)
Age, y	
<60	7
>60	17
Anatomic site of primary	
Oral Cavity	15
Oropharyngeal	9
Tumour Size	
T1	3
T2	10
T3	7
T4	4
Nodal spread	
N0	12
N1	0
N2a-c	12
Distant metastases	
M0	24
M1	0
HPV status	
HPV-positive	12
HPV-negative	12
CTC status	
CTC-positive	13 (Range from 1–12 CTCs/10 ml whole blood)
CTC-negative	11

**Table 2:** Clinicopathological findings including CTC status and Lung findings.

Patient	Gender	Age	HPV status	T Staging	N Staging	Description	Lung findings	CTC findings	CTC description
Micro_01	m	66	positive	T2	N0	Oral Cavity	lesion, query	Positive	1 CTC cluster ~ 12cells
Micro_02	m	54	unknown	T1	N0	Oropharyngeal		Negative	
Micro_03	m	63	negative	T4	N2c	Oropharyngeal	lesion, query	Positive	1 CTC
Micro_04	m	58	positive	T3	N2b	Oropharyngeal		Negative	
Micro_05	m	62	Positive	T2	N2c	Oropharyngeal		Negative	
Micro_06	m	42	negative	T3	N2	Oral Cavity		Positive	1 CTC cluster ~5 cells, 27 nucleated single cells
Micro_07	f	72	unknown	T1	N0	Oral Cavity		Negative	
Micro_08	m	48	unknown	T1	N0	Oral Cavity		Negative	
Micro_09	m	63	positive	T3	N2c	Oral Cavity		Positive	1 CTC cluster ~ 6 cells
Micro_10	m	61	positive	T2	N0	Oral Cavity		Negative	
Micro_11	m	73	unknown	T2	N0	Oral Cavity		Positive	1 CTC cluster ~ 5 cells
Micro_12	m	36	negative	T4	N2b	Oral Cavity	lesion, query	Positive	3 CTCs
Micro_13	m	56	positive	T2	N2b	Oropharyngeal		Positive	2 CTCs
Micro_14	f	55	positive	T4	N2b	Oropharyngeal		Positive	1 CTC
Micro_15	m	71	negative	T2	N0	Oral Cavity		Negative	
Micro_16	m	65	negative	T3	N2b	Oral Cavity		Positive	1 cluster ~ 5 cells + 2 single cells
Micro_17	m	60	Positive	T3	N2b	Oropharyngeal		Positive	1 cluster ~7 cells + 4 single cells
Micro_18	m	67	positive	T3	N0	Oral Cavity		Negative	
Micro_19	m	60	positive	T2	N2b	Oropharyngeal		Negative	
Micro_20	m	68	positive	T4	N0	Oral Cavity		Negative	6 CTCs
Micro_21	m	62	negative	T2	N0	Oropharyngeal		Negative	
Micro_22	m	68	negative	T3	N2b	Oral cavity		Positive	2 CTCs
Micro_23	m	89	negative	T2	N0	Oral Cavity		Negative	
Micro_24	m	60	negative	T2	No	Oral Cavity		Positive	3 CTCs
